# Genetic Alterations in Atypical Cerebral Palsy Identified Through Chromosomal Microarray and Exome Sequencing

**DOI:** 10.3390/ijms26072929

**Published:** 2025-03-24

**Authors:** Ji Yoon Han, Jin Gwack, Jong Hun Kim, Min Kyu Park, Joonhong Park

**Affiliations:** 1 Department of Pediatrics, College of Medicine, The Catholic University of Korea, Seoul 06591, Republic of Korea; han024@catholic.ac.kr; 2 Department of Pediatrics, Daejeon St. Mary’s Hospital, Daejeon 34943, Republic of Korea; 3 Department of Preventive Medicine, Jeonbuk National University Medical School, Jeonju 54907, Republic of Korea; gwackjin@jbnu.ac.kr; 4 Research Institute of Clinical Medicine of Jeonbuk National University-Biomedical Research Institute of Jeonbuk National University Hospital, Jeonju 54907, Republic of Korea; 5 Department of Thoracic and Cardiovascular Surgery, Jeonbuk National University Medical School and Hospital, Jeonju 54907, Republic of Korea; 41886@jbnu.ac.kr; 6 Department of Clinical Pharmacology and Therapeutics, Chungbuk National University College of Medicine and Hospital, Cheongju 28644, Republic of Korea; 7 Research Institute of Cheongju-Osong Advanced Clinical Trial Center, Osong 28161, Republic of Korea; 8 Department of Laboratory Medicine, Jeonbuk National University Medical School and Hospital, Jeonju 54907, Republic of Korea

**Keywords:** atypical cerebral palsy, chromosomal microarray, exome sequencing, genome sequencing, genetic diagnosis

## Abstract

This study investigated the genetic causes of atypical cerebral palsy (CP) through chromosomal microarray (CMA) and exome sequencing (ES) in a cohort of 10 Korean patients to identify variants and expand the spectrum of mutations associated with atypical cerebral palsy. Whole ES and/or genome sequencing (GS) after routine karyotyping and CMA was performed to identify causative variants and expand the spectrum of mutations associated with atypical CP. In cases of atypical CP, scoliosis and/or kyphosis, ranging from mild to severe, were present in all patients. Epilepsy was a comorbidity in seven patients (70%), and intellectual disability (ID) was observed in varying degrees. This study identified three copy number variations (CNVs), including 15q11.2 microdeletion (*n* = 1), 17p11.2 duplication (*n* = 1), and 12p13.33p11.23 duplication/18p11.32 microdeletion (*n* = 1), and six likely pathogenic variants (LPVs) or pathogenic variants (PVs) detected in the *SLC2A1*, *PLAA*, *CDC42BPB*, *CACNA1D*, *ALG12*, and *SACS* genes (*n* = 6). These findings emphasize the significance of incorporating genetic testing into the diagnostic process for atypical CP to improve our understanding of its molecular basis and inform personalized treatment strategies. To further advance this research, future studies should focus on exploring genotype–phenotype correlations, assessing the functional impact of identified variants, and increasing the sample size to validate the observed patterns.

## 1. Introduction

Cerebral palsy (CP) encompasses a group of lifelong disorders that impair movement and posture, leading to activity limitations [1]. These conditions result from non-progressive disruptions in the developing brain of a fetus or infant [2]. In addition to motor impairments, individuals with CP often experience challenges related to sensation, perception, cognition, communication, and behavior. Epilepsy and secondary musculoskeletal complications are also frequently observed [3]. During the 1980s and 1990s, the prevalence of CP in high-income countries was estimated at 2.1 per 1000 live births [4]. However, since 2010, this rate has declined significantly to 1.6 per 1000 live births, encompassing both pre/perinatal and postneonatal cases [5]. In contrast, low- and middle-income countries continue to report higher prevalence rates—up to 3.4 per 1000 live births—highlighting disparities in healthcare access and socio-economic conditions. CP prevalence is inversely related to gestational age and birthweight, with approximately 10% of cases classified as postnatal, mainly due to central nervous system (CNS) infections and head trauma [2,6]. CP arises from a combination of genetic, prenatal, perinatal, and postnatal influences. Major risk factors include genetic mutations, intrauterine infections, fetal growth restriction, preterm birth, and placental dysfunction [3]. Environmental factors and abnormal inflammatory responses may also interact with genetic predispositions, contributing to CP development [2].

Advances in genetic testing have deepened the understanding of CP’s genetic basis, challenging the conventional belief that perinatal complications are its primary cause [7]. Recent studies have provided greater insight into the etiology of CP, emphasizing the significant role of genetic factors in its development [8]. Among these, inborn errors of metabolism (IEM) and rare monogenic disorders can manifest with CP-like symptoms. Since many IEMs are treatable, early identification is crucial for timely intervention [9]. Other genetic contributors to CP include various monogenic syndromes, chromosomal abnormalities, and methylation defects [8]. Increasing evidence indicates that many atypical CP cases are linked to genetic abnormalities affecting neuronal development, synaptic function, and motor control pathways [3]. The implementation of next-generation sequencing (NGS) technologies has facilitated the identification of both known and novel genetic contributors to CP [10]. These technological advancements enhance diagnostic precision and offer new insights into disease mechanisms, paving the way for precision medicine strategies and improved prognostic guidance for patients and their families. Advancements in exome sequencing (ES) and genome sequencing (GS) have accelerated the discovery of genetic factors associated with neurodevelopmental disorders, including CP. ES, in particular, is an effective tool for detecting both novel and known pathogenic variants linked to rare diseases [8,11]. When combined with deep phenotyping, ES has achieved diagnostic success rates of 68–89% in well-characterized cohorts of individuals with suspected monogenic neurodevelopmental disorders [12]. These techniques have also been applied to CP, with a recent study identifying likely pathogenic variants in 9 of 17 cases across eight different genes, yielding a diagnostic success rate of 50%.

In this study, we investigated the genetic characteristics of atypical CP in a cohort of 50 Korean CP patients. These cases exhibited CP-like symptoms but lacked perinatal risk factors or the typical brain lesions associated with CP. Our analysis focused on both genetic factors and clinical manifestations. To identify genetic variants and expand the spectrum of mutations linked to atypical CP, we performed chromosomal microarray analysis (CMA) and ES.

## 2. Results

**Figure 1:**
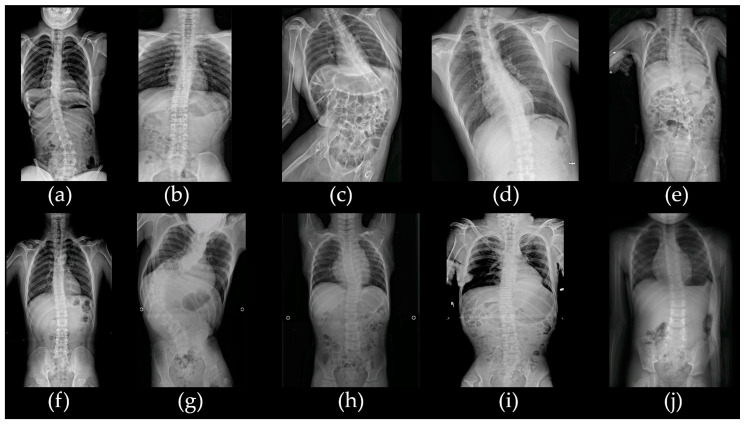
Radiologic findings demonstrate scoliosis in all patients with atypical cerebral palsy. (**a**) Patient acp01k, (**b**) patient acp02s, (**c**) patient acp03k, (**d**) patient acp04m, (**e**) patient acp05y, (**f**) patient acp06l, (**g**) patient acp07l, (**h**) patient acp08p, (**i**) patient acp09k, and (**j**) patient acp10k.

**Figure 2:**
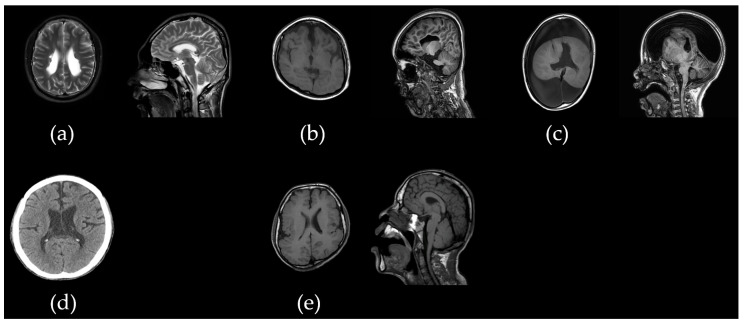
Brain magnetic resonance imaging features of atypical cerebral palsy. (**a**) Patient acp02s showed periventricular irregularities with associated hyperintensities on T2WI. (**b**) Patient acp03k showed frontotemporal atrophy with periventricular ischemic or demyelinating changes. (**c**) Patient acp05y showed hydranencephaly and lobal holoprosencephaly with open-lip schizencephaly with associated callosal agenesis and cortical malformation. (**d**) Patient acp08p showed widening of the side ventricles. (**e**) Patient acp09k showed schizencephaly with cortical dysplasia.

Among the 50 patients with CP, 10 were classified as having atypical CP. The cohort consisted of six males (60%) and four females (40%). Seven of these patients (70%) were diagnosed with spastic quadriplegia, two (20%) with spastic diplegia, and one (10%) with the athetoid type. Scoliosis and/or kyphosis, ranging from mild to severe, were present in all patients (Figure 1). Assessment of motor function using the Gross Motor Function Classification System (GMFCS) revealed that one patient (10%) was classified as grade II, one (10%) as grade III, and five (50%) as grade V. Epilepsy was a comorbidity in seven patients (70%), and intellectual disability (ID) was observed in varying degrees: profound in six patients (60%), severe in two (20%), mild in one (10%), and absent in one (10%). Five patients (50%) experienced hypotonia during the neonatal period, while congenital anomalies were found in two patients (20%) and sensorineural hearing loss (SNHL) in one patient (10%). Brain magnetic resonance imaging (MRI) showed that half of the 10 patients were normal, and the other half were abnormal (Figure 2). A family history of neurological disorders, such as ID or epilepsy, was reported in four patients (40%), and none of the families reported consanguinity. Genetic diagnoses were obtained for three patients through chromosomal analysis and CMA. The demographic and clinical characteristics of patients with atypical CP are summarized in Table 1.

**Figure 3:**
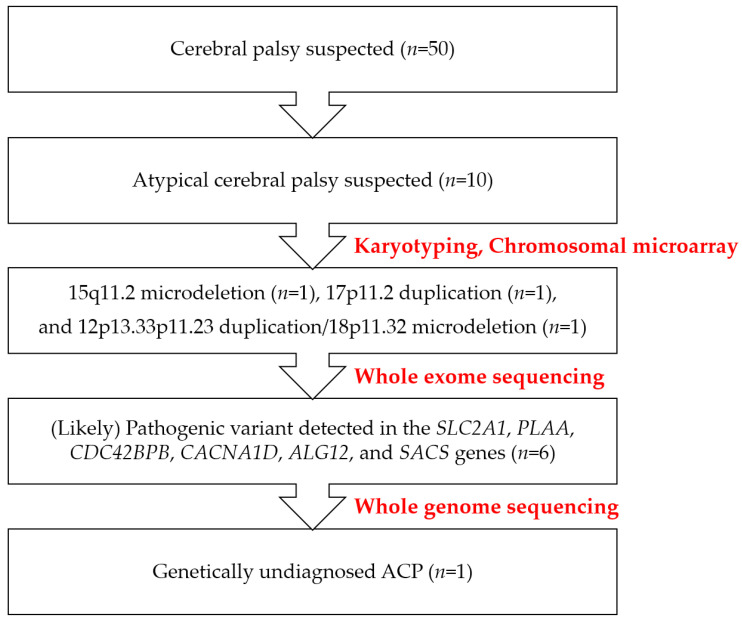
Diagnostic workflow for genetic diagnosis of atypical cerebral palsy (ACP).

WES was performed on six patients, leading to additional diagnoses, while WGS was also used, but one patient remained undiagnosed despite extensive genetic evaluation. Figure 3 depicts the diagnostic process and genetic testing results for this cohort. In cases of atypical CP, this study identified three copy number variations (CNVs), including 15q11.2 microdeletion (*n* = 1), 17p11.2 duplication (*n* = 1), and 12p13.33p11.23 duplication/18p11.32 microdeletion (*n* = 1), and six likely pathogenic variants (LPVs) or pathogenic variants (PVs) detected in the *SLC2A1*, *PLAA*, *CDC42BPB*, *CACNA1D*, *ALG12*, and *SACS* genes (*n* = 6). The genetic alteration profiles of the positive patients with atypical cerebral palsy and their clinical implications are summarized in Table 2.

### 2.1. Presentation of a Genetically Diagnosed Case Series in Atypical Cerebral Palsy

#### 2.1.1. Patient acp01k with *SLC2A1* c.277C>T/p.Arg93Trp Variant

The patient was born at 38 weeks of gestation via normal spontaneous delivery (NSD), with a birth weight of 3 kg. During the neonatal period, she exhibited generalized hypotonia, which persisted into infancy. Around 1 month of age, she developed generalized clonic seizures, requiring treatment with phenobarbital and phenytoin. As she grew, she showed global developmental delay (GDD), feeding difficulties, and intermittent movement abnormalities, including dystonic posturing. Spasticity progressively worsened after infancy, eventually leading to spastic quadriplegia and a diagnosis of cerebral palsy. By age 12, she had profound ID and severe motor impairment, classified as GMFCS grade V. Microcephaly was noted, with a head circumference below the third percentile. Brain MRI findings were normal, and an interictal sleep electroencephalogram (EEG) did not show any epileptiform discharges. Given the presence of early-onset seizures, developmental delay, microcephaly, and progressive spasticity, genetic testing was performed, revealing a pathogenic variant in *SLC2A1*, confirming a diagnosis of GLUT1 deficiency syndrome (GLUT1-DS). A cerebrospinal fluid (CSF) analysis showed markedly low glucose levels (hypoglycorrhachia), further supporting the diagnosis.

#### 2.1.2. Patient acp02s with 15p11.2 Microdeletion

Born at 29+5 weeks of gestation via cesarean section, he weighed 1.42 kg. Brain MRI revealed periventricular leukomalacia (PVL) grade II (Figure 2a). He demonstrated significant GDD, followed by profound ID and spastic quadriplegia, classified as GMFCS grade V. At the age of 20, he experienced a generalized tonic–clonic seizure and was prescribed anti-seizure medication (levetiracetam). The correlation between his brain lesions and clinical presentation revealed disproportionately severe cognitive and motor impairments. Additionally, the onset of epilepsy in early adulthood prompted genetic testing to identify the underlying disease.

#### 2.1.3. Patient acp03k with Bi-Allelic *PLAA* c.1039+1G>A and c.1834C>T/p.Pro612Ser Variants

She was born at 38 weeks of gestation via NSD, weighing 3 kg, with no significant perinatal events. However, from infancy, she exhibited GDD. She was unable to walk and could not speak a single meaningful word, presenting with profound ID and spastic quadriplegia. Her GMFCS grade was V. Seizures began around the age of 2, and she has been on valproic acid since. Brain MRI revealed both frontotemporal atrophy and periventricular demyelinating changes (Figure 2b). EEG findings showed focal sharp waves in both temporal regions, along with a generally slow background pattern.

#### 2.1.4. Patient acp05y with *CDC42BPB* c.4049G>A/p.Arg1350Gln Variant

He was born at 36 weeks of gestation via NSD, weighing 2.5 kg. His neonatal period was unremarkable, but he later exhibited GDD. Spasticity became evident during his toddler years, and seizures began around the age of 2 months. He has been on valproic acid since then. He presents with profound ID, spastic quadriplegia, and a GMFCS grade of V. Brain MRI revealed lobar holoprosencephaly with open-lip schizencephaly and cortical malformation (Figure 2c). EEG findings showed multifocal epileptiform discharges.

#### 2.1.5. Patient acp06l with 17p11.2 Duplication

He was born at 40 weeks of gestation via cesarean section, weighing 3.36 kg. During the neonatal period, he exhibited mild hypotonia, which progressed to spasticity in the lower limbs as he entered infancy, along with GDD. In childhood, he presented with severe ID, spastic diplegia, and a GMFCS grade of IV. Brain MRI findings were normal, and he did not experience seizures.

#### 2.1.6. Patient acp07l with *CACNA1D* c.1846T>C/p.Cys616Arg Variant

He was born at 37 weeks of gestation via NSD, weighing 2.9 kg. During the neonatal period, he exhibited hypotonia, followed by GDD and the development of spasticity, leading to a diagnosis of CP. Brain MRI findings were unremarkable, but he presented with profound ID and a GMFCS grade of V. Seizures began during adolescence and were eventually controlled after trials of multiple antiepileptic drugs. He is currently on levetiracetam and perampanel. Seizures worsened with the use of valproic acid, lamotrigine, and clobazam, which were discontinued. Vital signs and blood tests, including electrolyte levels, remained consistently within normal ranges.

#### 2.1.7. Patient acp08p with Bi-Allelic *ALG12* c.437G>A/p.Arg146Gln and c.788A>G/p.Tyr263Cys Variants

He was born at 33 weeks of gestation via NSD, weighing 2.56 kg. Severe hypotonia was evident at birth, and due to failure to thrive, he required tube feeding until the age of 6 months. Tongue hypotonia caused significant swallowing difficulties, which led to a reduction surgery at 5 months of age. Clinical examination revealed facial dysmorphism, thin and long fingers, cryptorchidism, soft tissue laxity, and clubfoot. Echocardiography showed mild aortic dilatation without other structural abnormalities. Brain MRI revealed enlarged bilateral ventricles, but no additional structural anomalies were identified (Figure 2d). Blood tests showed mildly elevated liver enzymes, while immune and coagulation profiles were within normal ranges. Despite generalized hypotonia, joint contractures were present. He exhibited profound ID, spastic quadriplegia, and a GMFCS level of V.

#### 2.1.8. Patient acp09k with 12p13.33p11.23 Duplication and 18p11.32 Microdeletion

He was born at 41 weeks of gestation via NSD, weighing 3 kg. During the neonatal and infantile periods, he exhibited mild hypotonia and GDD. He was diagnosed with a cleft palate and underwent corrective surgery at 12 months of age. As he entered the toddler period, spasticity became more prominent, and he developed profound ID and spastic quadriplegia and was classified as GMFCS grade V. Around the age of 15, he began experiencing myoclonic astatic seizures, which were successfully managed with levetiracetam. Brain MRI revealed schizencephaly with cortical dysplasia (Figure 2e), and EEG findings showed sharp waves in both temporal regions, along with generalized background slowing. Blood tests indicated leukopenia (3300/μL) and thrombocytopenia (98 K/μL), which are currently being monitored.

#### 2.1.9. Patient acp10k with Bi-Allelic *SACS* c.11101T>C/p.Trp3701Arg and c.12973C>T/p.Arg4325Ter Variants

She was born at 39 weeks of gestation via NSD, weighing 3.5 kg, with no significant abnormalities noted during the early infantile period. However, delayed motor development became evident in late infancy, and by early childhood, she developed an ataxic gait with frequent imbalance and coordination difficulties. Her symptoms progressively worsened, and by the age of 12, she began experiencing frequent falls despite being able to walk independently, eventually requiring the use of assistive devices. As the disease progressed, her motor function further declined, and by adolescence, she lost the ability to walk independently and now requires a wheelchair for mobility. She was also diagnosed with bilateral SNHL and mild ID. Seizures first occurred at the age of 7, presenting as generalized tonic seizures, which were effectively controlled with valproic acid and levetiracetam. Neuroimaging findings showed a progressive neurodegenerative pattern. A brain MRI performed at 2 years of age appeared normal, but a follow-up MRI at 12 years revealed diffuse brain atrophy, particularly affecting the cerebellum and brainstem, consistent with neurodegenerative disease progression. EEG findings indicated epileptiform discharges in the central areas, with secondary generalization.

## 3. Discussion

CP is a broad term that encompasses a group of permanent disorders affecting movement and posture development, resulting in activity limitations. These issues are linked to non-progressive disturbances in the developing fetal or infant brain. CP is typically classified based on motor signs (including spasticity, dyskinesia, ataxia, and hypotonia—spasticity is seen in about 80% of cases), the extent of limb involvement (hemiplegia, monoplegia, diplegia, triplegia, and quadriplegia), and the anatomical location of brain lesions (cerebral cortex, pyramidal tract, extrapyramidal system, and cerebellum) [7,13]. While motor impairments are central to CP, over half of patients also experience additional comorbidities, such as epilepsy, language impairments, ID, autism spectrum disorder (ASD), and visual or hearing deficits, highlighting its classification as a complex neurodevelopmental disorder [14]. Muscle tone, posture, and movement alterations often manifest in early childhood due to disrupted brain development, typically following a permanent and non-progressive course. These motor issues often co-occur with other neurodevelopmental disorders, including ID (27–45%), epilepsy (38%), speech disorders (33–82%), and ASD (3–9%) [15,16]. These comorbidities not only complicate clinical features but also emphasize the need for comprehensive evaluations to determine the underlying etiology. In our study, patients with atypical CP received standard rehabilitation therapies, including physical and occupational therapy, similar to those with typical CP. There were no significant differences in motor rehabilitation approaches between the two groups. While the degree of improvement varied, treatment outcomes depended on individual severity and comorbidities. In genetically diagnosed cases, specific management strategies were considered, but the overall rehabilitation approach remained consistent. MRI scans reveal abnormalities in approximately 85% of children with CP [17], and MRI is valuable for estimating lesion timing and assessing whether the lesion contributes to motor impairment or is an incidental finding [18]. If MRI results are normal, further investigation into potential genetic conditions, such as hereditary spastic paraplegias, dopamine-responsive dystonia, or metabolic disorders that mimic CP, may be necessary.

Genetic testing offers psychological reassurance by providing a definitive diagnosis, minimizing uncertainty, and helping families comprehend the patient’s prognosis [19]. Identifying a genetic cause enables more effective medical planning and facilitates access to personalized support, thereby reducing caregiver stress [20]. Integrating genetic testing into clinical practice not only optimizes medical management but also contributes to the emotional well-being of both patients and caregivers [21]. In our study, the use of multiple genetic diagnostic tools, including CMA and ES/GS, allows for a thorough investigation of potential genetic causes. This is particularly relevant given the complex and heterogeneous nature of atypical CP. We observed genetic heterogeneity, with chromosomal abnormalities including deletions and duplications such as 15p11.2 deletion, 17p11.2 duplication, 12p13.33-p11.23 duplication, and 18p11.32 deletion. The 15q11.2 microdeletion syndrome, a rare partial autosomal monosomy, is characterized by variable phenotypic expression and reduced penetrance [22]. This syndrome is linked to a heightened risk of neuropsychiatric and neurodevelopmental disorders, including psychomotor and speech delays, ASD, attention-deficit/hyperactivity disorder (ADHD), obsessive–compulsive disorder, epilepsy, and seizures [23]. Hypotonia in infancy is commonly reported, which may later evolve into spasticity [22]. Although the patient acp02s in our study showed PVL due to preterm birth, the 15p11.2 deletion may account for the profound ID, likely contributing to the more severe developmental delays and motor impairments observed. Patients with 17p11.2 duplication syndrome, also known as Potocki–Lupski syndrome (PTLS), often present with mild to moderate ID, speech and language delays, and hypotonia in infancy, which may progress to spasticity in some cases [24]. Behavioral features include ASD, ADHD, and anxiety, while physical features may include distinctive craniofacial characteristics, feeding difficulties, and congenital heart defects such as atrial or ventricular septal defects [25]. The duplication involves several genes, with RAI1 being the most critical. RAI1 is a transcriptional regulator, and its dosage imbalance is linked to many of the neurodevelopmental and behavioral features seen in PTLS [26]. Other genes in this region may contribute to variability in the phenotype. The 12p13.33-p11.23 duplication spans approximately 27.5 Mb on the short arm of chromosome 12 and includes several key genes related to neurodevelopment and cellular function. Notable genes such as *PAFAH1B1*, involved in neuronal migration, are associated with cortical malformations like schizencephaly and developmental delays [27]. *ETV6* and *CDKN1B*, linked to cell cycle regulation and hematologic disorders, may also contribute to additional systemic manifestations [28]. Duplications in 12p are often associated with ID, motor delays, and features resembling cerebral palsy, depending on gene dosage effects [29]. The 18p11.32 deletion spans about 0.9 Mb on the short arm of chromosome 18 and includes essential genes like *USP14*, which is critical for protein degradation through the ubiquitin–proteasome system [30]. Disruption of *USP14* can lead to profound ID, spasticity, hypotonia, and neurodevelopmental delay (NDD). Additional symptoms associated with deletions in this region may include epilepsy, motor deficits, and structural brain abnormalities, such as ventriculomegaly or schizencephaly [31,32]. This deletion is strongly linked to neurodevelopmental disorders due to its impact on critical neuronal pathways. The presence of two CNVs may have an additive or synergistic effect, worsening the overall phenotype.

On the other hand, ES is now recommended as a first-tier clinical diagnostic test for individuals with NDDs [19]. Across multiple studies, the average molecular diagnostic yield of ES for various NDDs has been reported as 35% for ID or NDD, 45% for epilepsy, and 15% for ASD, all of which are commonly associated with CP [33,34,35]. Since CP is often diagnosed earlier than other NDDs, such as ID or ASD, early genetic testing in CP patients could lead to a high diagnostic yield and should be strongly considered. A review of inborn errors of metabolism that present with CP-like symptoms identified 54 treatable and 43 non-treatable metabolic conditions that can mimic or present as CP [9]. In our study, we also identified a treatable metabolic disorder—GLUT1-DS. Determining the genetic causes of CP not only provides families with a definitive diagnosis but also aids in informed family planning, connects them to condition-specific resources, and contributes to gene-specific research. Additionally, genomic-guided interventions and surveillance have led to improved medical management in 36.8% of individuals with pathogenic or likely pathogenic (P/LP) variants [21]. ES has been shown to identify a genetic cause in 15% to 34% of CP cases, and commercial gene panels specifically designed for CP are now clinically available [36]. The genetic landscape of CP is highly diverse, including numerous rare single-gene mutations such as *AGAP1*, *AMPD2*, *AP4M1*, *ATL1*, *CACNA1A*, *COL4A1*, *CTNNB1*, *FBXO31*, *ITPR1*, *KDM7A*, *KIF1A*, *MAOB*, *NT5C2*, *RHOB*, *SCN2A*, *SPAST*, *STXBP1*, and *TUBA1A*. Additionally, several recurrent likely pathogenic CNVs have been identified, including dup Xp22.33, del Xp22.33, dup 2p25.3 (*MYT1L*), del 9p22.53 (*KANK1*), and del 22q11.21 [20]. Given the increasing use of genetic testing and the growing number of CP-associated genes being discovered, it is essential to evaluate how these findings impact the clinical care and management of individuals with CP [37]. Our study further highlights the wide range of genes implicated in atypical CP presentations, including *SLC2A1*, *PLAA*, *CDC42BPB*, *CACNA1D*, *ALG12*, and *SACS*. Detailed clinical features and demographic information for the patients provide context for the genetic findings. This could be valuable for clinicians trying to make informed decisions based on similar clinical presentations. In detail, the *SLC2A1* gene encodes GLUT1, a glucose transporter protein that facilitates glucose transport across the blood–brain barrier and into cells [38]. GLUT1-DS can present with symptoms resembling CP, including early-onset spasticity, developmental delay, and epilepsy, which may lead to misdiagnosis. A key distinguishing feature is CSF hypoglycorrhachia, indicating impaired glucose transport across the blood–brain barrier due to *SLC2A1* mutations. Unlike CP, GLUT1-DS is treatable with a ketogenic diet, making early diagnosis crucial. Given the growing recognition of genetic contributions to CP-like phenotypes, metabolic and genetic testing should be considered in atypical CP cases to identify treatable conditions [39]. The phenotypic spectrum of GLUT1-DS varies widely, ranging from severe encephalopathy in infancy to isolated paroxysmal exercise-induced dyskinesia in later life [40]. Clinical manifestations may include ID, microcephaly, dystonia, chorea, ataxia, spasticity, and various paroxysmal events such as seizures and paroxysmal dyskinesia. In our study, an initial CP diagnosis resulted in a significant delay in identifying the underlying condition, which was ultimately confirmed as GLUT1-DS—a treatable disorder.

The *PLAA* gene encodes phospholipase-A2-activating protein, which plays a critical role in ubiquitin-mediated endolysosomal degradation of proteins—an essential process for maintaining synaptic and cellular homeostasis [41,42]. Additionally, it is involved in prostaglandin synthesis and cytoskeletal organization, both of which influence neural development and function. Mutations in the *PLAA* gene cause neurodevelopmental disorder with progressive microcephaly, spasticity, and brain anomalies (NDMSBA; OMIM #617527), a rare autosomal recessive condition. Individuals with *PLAA* mutations typically present with progressive microcephaly, profound GDD, and severe spastic quadriparesis, often accompanied by dystonia [43,44,45]. Neurological symptoms frequently include early-onset seizures, while brain imaging commonly reveals delayed myelination, a thin corpus callosum, and progressive cerebral or cerebellar atrophy.

*CDC42BPB* is a member of the serine/threonine kinase family and encodes myotonic-dystrophy-related Cdc42-binding kinase beta (MRCKβ). This protein plays a vital role in cytoskeletal reorganization and cell migration in non-muscle cells by phosphorylating the myosin II regulatory light chain (MLC2) [46]. Individuals with *CDC42BPB* mutations exhibit severe neurodevelopmental impairments, including GDD and motor dysfunction. Other key features include early-onset epilepsy, spasticity, and profound ID. Brain imaging often reveals structural abnormalities such as polymicrogyria, schizencephaly, or other cortical malformations [47]. In our case, the patient exhibited holoprosencephaly along with severe GDD and epilepsy, suggesting the possibility of an additional contributing genetic or environmental factor. Given the expanding phenotypic spectrum of *CDC42BPB*-related disorders, further studies with larger cohorts are necessary to determine whether holoprosencephaly is a rare manifestation or an incidental finding unrelated to the mutation. Regarding *CDC42BPB* mutations, cases with cystic changes in the brain have been reported; however, to our knowledge, no cases presenting with holoprosencephaly have been documented.

The *CACNA1D* gene encodes a subunit of the L-type voltage-gated calcium channel (Cav1.3), which is crucial for calcium influx into cells in response to membrane depolarization [17,48]. This channel plays an essential role in various physiological processes, including neural activation, synaptic transmission, hormone secretion, and cardiac and auditory functions [49]. While some *CACNA1D* mutations are linked to primary aldosteronism, others are associated solely with neurological manifestations without affecting aldosterone regulation [17]. The variability in clinical presentation is likely due to the specific nature and location of the mutations, which influence calcium channel function in different tissues. In the brain, *CACNA1D* is vital for neuronal excitability, synaptic plasticity, and the regulation of calcium-dependent signaling pathways. Mutations or dysfunctions in this gene have been implicated in several neurological and psychiatric conditions, including neurodevelopmental disorders such as DD, ID, and ASD, as well as epilepsy, schizophrenia, mood disorders, and movement disorders [17,50].

The *ALG12* gene encodes α-1,6-mannosyltransferase, an enzyme crucial for proper N-glycan synthesis during protein glycosylation in the endoplasmic reticulum [51]. This enzyme facilitates the addition of the eighth mannose residue to the growing glycan chain, a critical step in the assembly of dolichol-linked oligosaccharides. Mutations in *ALG12* disrupt this process, leading to defective glycosylation and causing congenital disorder of glycosylation type Ig (CDG-Ig), a multisystemic condition characterized by GDD, ID, hypotonia, seizures, and failure to thrive [52]. Additional clinical features may include hepatic dysfunction (elevated liver enzymes or coagulopathy), gastrointestinal issues (chronic diarrhea or feeding difficulties), and endocrine abnormalities such as hypoglycemia [53]. In some cases, patients may also exhibit skeletal dysplasia, cardiac defects, or immune dysfunction [54].

The *SACS* gene encodes sacsin, a large protein essential for maintaining mitochondrial function and cytoskeletal organization, particularly in neurons. Sacsin plays a key role in protein quality control and stabilizing microtubules, both of which are crucial for neuronal health and function [55]. Mutations in *SACS* cause spastic ataxia of Charlevoix–Saguenay, a rare autosomal recessive neurodegenerative disorder characterized by early-onset spasticity, progressive cerebellar ataxia, and peripheral neuropathy [56,57]. Additional clinical features may include dysarthria, distal muscle wasting, and retinal hypermyelination. As the disease progresses, patients often experience a decline in motor function, eventually requiring mobility aids or wheelchairs. Brain MRI findings typically include atrophy of the superior cerebellar peduncles, pontocerebellar atrophy, and a characteristic double-line sign in the pons [58]. Other findings may involve corpus callosum thinning, mild cerebral atrophy, and T2 hyperintensities in the periventricular white matter, reflecting the widespread neurodegeneration associated with *SACS* mutations.

With the increasing availability of genomic tools for genetic diagnosis, it has become evident that atypical CP often has a genetic origin [36,59,60,61,62,63,64,65,66] (Table 3). The highest diagnostic yields have been observed in cohorts focusing on patients with CP who were born at full term, had no significant abnormalities on brain MRI, or exhibited atypical CP features suggestive of a genetic cause. Such features include brain malformations, syndromic disorders affecting the CNS, motor dysfunction, and abnormal neurodevelopment during infancy and early childhood. These conditions are often classified as CP mimics or congenital idiopathic CP [9,67]. Our study applied strict inclusion and exclusion criteria, with the diagnostic process determined collaboratively by pediatric neurologists, rehabilitation physicians, and clinical geneticists. This multidisciplinary approach likely contributed to the higher diagnostic yield (90%) observed in our cohort. Among our patients, 50% had normal MRI findings, and epilepsy was present in 60% of cases. Additionally, 50% exhibited neonatal hypotonia, which progressed to spasticity, followed by DD and ID as they aged. When considering genetic testing, several phenotypic categories are particularly relevant, including dyskinesia, absence of spasticity, ID, or ASD, term birth with normal brain MRI findings, cognitive impairment and communication difficulties, parental consanguinity, and progressive neurological symptoms [36,59,60,61,62,63,64,65,66]. Atypical CP presents with distinct features that differentiate it from classic CP, serving as diagnostic clues that warrant further etiological investigation. By applying rigorous selection criteria, we were able to identify genetic causes in a substantial proportion of cases. However, many existing studies are limited to case reports or include patients who do not align with current consensus criteria for CP, making interpretation challenging [68]. The lack of a standardized definition for cryptogenic, idiopathic, or atypical CP, along with no consensus guidelines on which CP patients should undergo genetic evaluation, underscores the urgent need to revise diagnostic criteria and clinical practice parameters. Incorporating genetic testing into routine diagnostic protocols would enhance classification and improve diagnostic accuracy in the evolving genetic landscape of CP. 

Our study has several limitations. First of all, while the study included 50 patients with CP, only 10 were classified as having atypical CP. This limited sample size for the subgroup of interest (atypical CP) makes it challenging to generalize the findings to a broader population. A larger cohort would improve the robustness of the conclusions and provide more statistical power to detect trends or correlations. Second, despite the extensive genetic testing methods employed, one patient remained undiagnosed. While this may be due to the limits of current genetic testing technology or the presence of rare mutations, it highlights the incomplete diagnostic yield of genetic tests, especially for cases with more complex or novel genetic causes. Third, there are limited long-term follow-up data on the clinical outcomes of patients after the genetic diagnosis. Understanding how the genetic diagnosis influences long-term prognosis, clinical management, and therapeutic interventions is crucial. This would help assess the practical impact of genetic diagnosis on patient care. Fourth, while the study successfully identified known genetic mutations associated with CP, it did not delve deeply into the potential role of environmental factors, epigenetics, or interactions between genetic mutations and external factors (such as prenatal conditions). A more holistic approach that integrates genetic, environmental, and epigenetic data could provide more comprehensive insights into atypical CP. Fifth, the study could benefit from more detailed discussion on the implications of genetic findings for family counseling, recurrence risk, and family planning. While the study touches on the importance of genetic diagnosis for families, a more thorough analysis of how genetic testing influences family decision-making and patient management would enhance the clinical relevance of the research. Sixth, while WES and WGS offer substantial potential, the study does not provide a clear comparison of diagnostic yield between these methods. It would be beneficial to assess whether WES or WGS provides a higher diagnostic rate than CMA or chromosomal analysis, particularly for atypical CP cases. This would inform clinicians about which testing methods to prioritize based on available resources. Finally, while the study highlights the broad range of clinical presentations associated with various genetic mutations, there may be a lack of in-depth analysis of the phenotypic variability for certain mutations. For example, patients with the same genetic mutation (e.g., *SLC2A1* or *PLAA*) presented with different clinical features, which raises the question of incomplete penetrance or variable expressivity. Exploring this further would help in predicting outcomes based on genetic findings. However, it accurately reflects real-world clinical practice in a tertiary referral center and underscores the complexity of diagnosing CP. Identifying specific genetic causes not only facilitates etiologic treatments but also enhances the understanding and management of CP for patients, families, and healthcare providers. Further research is essential to clarify the clinical implications of genetic testing in CP and to optimize diagnostic and treatment strategies.

## 4. Materials and Methods

### 4.1. Patients

The cohort consisted of 10 patients with atypical CP who visited the Department of Pediatric Neurology at Daejeon St. Mary’s Hospital (Daejeon, Republic of Korea) between January 2020 and December 2024. The inclusion criteria were as follows: (1) normal brain MRI findings despite motor disabilities or a mismatch between perinatal history and MRI results; (2) severe motor symptoms or ID in the absence of a perinatal injury history; (3) a progressive or regressive disease course; and (4) the presence of congenital malformations or dysmorphic features. These inclusion criteria were applied using an ‘or’ condition, meaning that patients were included if they met at least one of the specified criteria (1, 2, 3, or 4), rather than requiring all conditions to be fulfilled simultaneously. Patients were excluded if they had (5) perinatal complications, such as asphyxia, respiratory distress syndrome requiring mechanical ventilation, or CNS infection, or (6) acquired brain MRI lesions, including hemorrhagic or ischemic damage. A comprehensive chart review was conducted, documenting perinatal and family history, developmental milestones, associated medical conditions, findings from general and neurological examinations, and functional status, assessed using the GMFCS. Disease progression was evaluated through detailed medical history and neurological assessments. Each patient underwent thorough evaluations by a pediatric neurologist, rehabilitation physician, and medical geneticist. Before genetic testing, a full biomedical and metabolic work-up was performed, including analyses of plasma amino acids, urine organic acids, thyroid function tests, lactate/pyruvate levels, blood gas, and urine oligosaccharides and mucopolysaccharides, as well as carnitine profiling.

### 4.2. Single Nucleotide Polymorphism (SNP) Microarray and Data Interpretation

Genomic DNA (gDNA) was extracted from whole blood samples of patients who underwent karyotyping. The extracted gDNA samples were analyzed at the Certified Analytics Professional (CAP)-certified GC Genome Laboratories (Yongin, Republic of Korea) using the Affymetrix CytoScan Dx Assay, a high-density CMA platform that integrates comparative genomic hybridization (CGH) and single nucleotide polymorphism (SNP) analysis. This assay evaluates approximately 2,696,550 markers, including up to 750,000 SNPs and 1.9 million non-polymorphic markers. The CytoScan Dx Assay was processed on the GeneChip System 3000Dx (Thermo Fisher Scientific, Waltham, MA, USA) following the manufacturer’s protocol. The resulting array was scanned using a GeneChip Scanner, and the signal intensity of each marker was assessed. Data analysis was performed using Chromosome Analysis Suite (ChAS Dx) software, version 3.1 (Thermo Fisher Scientific), comparing sample signals to a reference set derived from an average of over 400 samples. Differences in signal intensity between the sample and reference were expressed as a log_2_ ratio, representing the relative intensity of each marker. The filter settings for copy number variant (CNV) reporting were set at a minimum of 50 kb and 50 markers for copy number gains, and 25 kb and 25 markers for copy number losses. The relative intensity data were used to determine discrete copy number values, which were displayed accordingly. Genotype information for SNP markers was visualized using Allele Track.

Using Human Genome Build 19 (Genome Reference Consortium GRCh37), the CytoScan Dx Assay CNV coordinates were compared with actionable microarray findings listed in the following databases: ClinGen (https://clinicalgenome.org/; accessed on 13 October 2024); dbVar (https://www.ncbi.nlm.nih.gov/dbvar/; accessed on 13 October 2024); Decipher (https://www.deciphergenomics.org/browser; accessed on 13 October 2024); and Online Mendelian Inheritance in Man (OMIM) (https://www.omim.org/; accessed on 13 October 2024). The CMA results were exclusively interpreted by board-certified healthcare professionals specializing in molecular genetics or clinical cytogenetics. CNVs were classified into four categories based on the American College of Medical Genetics (ACMG) guidelines for postnatal constitutional CNV interpretation and reporting: pathogenic, variants of possible significance (VOPS), variants of unknown significance (VOUS), and benign. CNVs classified as pathogenic or VOPS were considered clinically significant [69].

### 4.3. Library Preparation, Exome Sequencing, Genome Sequencing, and Bioinformatic Analysis

The gDNA was extracted from peripheral blood samples using the QIAamp DNA Mini Kit, following the manufacturer’s protocol (Qiagen, Hilden, Germany). Whole ES/GS was performed at GC Genome (Yongin, Republic of Korea), a CAP-accredited clinical laboratory, for research purposes. Comprehensive singleton whole ES and/or whole GS were sequentially conducted on the proband from each family using the MGIEasy Exome Capture V5 Probe Set, which targets 22,000 genes, including mitochondrial DNA (mtDNA), and/or the MGIEasy PCR-Free DNA Library Prep Set (MGI Tech Co., Ltd., Shenzhen, Guangdong, China), following routine karyotyping and CMA, respectively. The enriched libraries were pooled and sequenced in parallel using the DNBSEQ-G400RS High-throughput Sequencing Set on the DNBSEQ-G400 for whole ES and/or the DNBSEQ-T7RS High-throughput Sequencing Set on the DNBSEQ-T7 sequencer for whole GS (MGI Tech Co., Ltd., Shenzhen, China).

Sequencing and bioinformatics analyses followed the Genome Analysis Tool Kit (GATK) Best Practices pipeline (https://gatk.broadinstitute.org/hc/en-us; accessed on 13 October 2024). This comprehensive workflow included base-calling, sequence alignment, variant calling, annotation, and quality control reporting. Sequences were aligned to the hg19 reference genome using BWA-aln, and single nucleotide variants (SNVs) as well as small insertions or deletions (indels) were identified using GATK version 4.5.0.0 with HaplotypeCaller and validated using DRAGEN v3.7.8. Variant interpretation adhered to the five-tier classification system established by the American College of Medical Genetics and Genomics (ACMG) and the Association for Molecular Pathology (AMP) [70]. Additionally, computational tools for assessing missense variant pathogenicity were applied in accordance with ClinGen recommendations for PP3/BP4 criteria [71]. Potentially pathogenic variants were identified based on the following filtering criteria: (1) variants with an allele frequency of less than 0.01; (2) variants located near or within exons of protein-coding genes associated with epilepsy and their clinical significance; (3) de novo or rare variants identified in the proband that were heterozygous, compound heterozygous, or homozygous within the same gene; (4) variants that resulted in nonsynonymous or nonsense codon changes, affected highly conserved splice sites, or caused frameshift mutations; (5) variant frequencies in the general population assessed using the Genome Aggregation Database (gnomAD) (https://gnomad.broadinstitute.org/; accessed on 13 October 2024). For all variants identified in the patients, we performed Sanger sequencing on trio samples (the proband and both parents) to confirm their presence and inheritance pattern. For variants detected only in the patient as de novo variants, the biological parentage of the patient’s parents was confirmed through multiplex short tandem repeat analysis using AmpFlSTR^®^ Identifiler™ amplification kit (Applied Biosystems, Foster City, CA, USA).

## 5. Conclusions

Atypical CP in this cohort was linked to a variety of genetic causes, and a combination of chromosomal analysis, CMA, WES, and WGS proved instrumental in identifying these genetic conditions. Despite the complexity and sometimes negative results (e.g., one undiagnosed patient after comprehensive genetic testing), these findings open the door to improved diagnostic strategies and better patient care for atypical CP. In particular, the identification of a range of genetic mutations (e.g., *SLC2A1*, *PLAA*, *CDC42BPB*, and others) in the cohort is valuable, as it enriches the understanding of atypical CP and its genetic etiology. The study’s contribution to identifying treatable conditions like GLUT1-DS is particularly important for clinical management and improved patient outcomes. These findings emphasize the significance of incorporating genetic testing into the diagnostic process for atypical CP to improve our understanding of its molecular basis and inform personalized treatment strategies. Further studies should focus on assessing the functional roles of these genes by utilizing knock-out or knock-in animal models to determine the extent of brain and motor function impairment. Additionally, investigating mutations at the cellular level could facilitate the growth of neurons, allowing researchers to study their abnormal functioning.

## Figures and Tables

**Table 1:** Clinical information of patients diagnosed with atypical cerebral palsy.

Patient	S/A (y)	GA/BW	CP Types	GMFCS	ID	Epi/OA	Other Comorbidities	Brain MRI
acp01k	F/13	38 wks/3 kg	SQ	V	Profound	+/1 mo	NH, microcephaly	Normal
acp02s	M/21	29+5 wks/1.42 kg	SQ	V	Severe	+/20 y	None	Periventricular leukomalacia (grade II)
acp03k	F/15	38 wks/3 kg	SQ	V	Profound	+/12 mo	None	Frontotemporal atrophy with demyelinating changes
acp04m	F/39	38 wks/3 kg	Athetoid	II	Normal	−/−	None	Normal
acp05y	M/9	36 wks/2.5 kg	SQ	V	Profound	+/2 mo	None	Schizencephaly with associated callosal agenesis, hydranencephaly
acp06l	M/18	40 wks/3.36 kg	SD	V	Severe	−/−	NH	Normal
acp07l	M/20	37 wks/2.9 kg	SQ	V	Profound	+/16 y	NH	Normal
acp08p	M/17	33 wks/2.56 kg	SQ	V	Profound	−/−	NH, cryptorchidism, facial dysmorphism, thin and long fingers	Widening of the side ventricles
acp09k	M/25	41 wks/3 kg	SQ	V	Profound	+/25 y	NH, cleft palate	Schizencephaly with cortical dysplasia
acp10k	F/8	39 wks/3.5 kg	SD	III	Mild	+/7 y	SNHL, ataxia	Normal

S/A (y), sex/age (year); GA/BW, gestational age/birth weight; wk, week; CP, cerebral palsy; SQ, spastic quadriplegia; SD, spastic diplegia; GMFCS, gross motor function classification system; ID, intellectual disability; Epi/OA, epilepsy/onset age; +, Positive; −, Negative; NH, neonatal hypotonia; mo, month; y, year; MRI, magnetic resonance imaging.

**Table 2:** Genetic alteration profiles of the positive patients with atypical cerebral palsy and their clinical implications.

Patient	Genetic Alteration	Origin/Zygosity	gnomAD	ACMG Class	OMIM Phenotype
acp01k	*SLC2A1*, c.277C>T/p.Arg93Trp	De novo/Het	n.f.	PV	GLUT1DS1 (# 606777)
acp02s	15q11.2 microdeletion	Mat/Het	n.f.	PV	CHR15q11.2DS (# 615656)
acp03k	*PLAA*, c.1039+1G>A *PLAA*, c.1834C>T/p.Pro612Ser	Pat/Het Mat/Het	n.f.	LPV	NDMSBA (# 617527)
acp05y	*CDC42BPB*, c.4049G>A/p.Arg1350Gln	De novo/Het	n.f.	LPV	CHOCNS (# 619841)
acp06l	17p11.2 duplication	Mat/Het	n.f.	PV	PTLS (# 610883)
acp07l	*CACNA1D*, c.1846T>C/p.Cys616Arg	De novo/Het	n.f.	LPV	PASNA (# 615474)
acp08p	*ALG12*, c.437G>A/p.Arg146Gln *ALG12*, c.788A>G/p.Tyr263Cys	Pat/Het Mat/Het	0.0113 0.0004	PV LPV	CDG1G (# 607143)
acp09k	12p13.33p11.23 duplication 18p11.32 microdeletion	De novo/Het De novo/Het	n.f.	LPV LPV	n.a.
acp10k	*SACS*, c.11101T>C/p.Trp3701Arg *SACS*, c.12973C>T/p.Arg4325Ter	Pat/Het Mat/Het	0.0008 0.0008	PV LPV	SACS (# 270550)

Het, heterozygous; Mat, maternal; Pat, paternal; gnomAD, The Genome Aggregation Database; n.f., not found; ACMG, American Medical College of Medical Genetics and Genomics; PV, pathogenic variant; LPV, likely pathogenic variant; OMIM, Online Mendelian Inheritance in Man; GLUT1DS1, GLUT1 deficiency syndrome 1, infantile onset, severe; CHR15q11.2DS, chromosome 15q11.2 deletion syndrome; NDMSBA, neurodevelopmental disorder with progressive microcephaly, spasticity, and brain anomalies; CHOCNS, Chilton–Okur–Chung neurodevelopmental syndrome; PTLS, Potocki–Lupski syndrome; PASNA, primary aldosteronism, seizures, and neurologic abnormalities; CDG1G, congenital disorder of glycosylation, type Ig; n.a., not available; SACS, spastic ataxia, Charlevoix–Saguenay type.

**Table 3:** Genetic results of patients with atypical cerebral palsy (CP) in previous reports.

References	Patients (n)	Diagnostic Yield (%)	Genetic Tests	Patients	Genetic Alterations	Remarks
Moreno-De-Luca et al. (2021) United States of America [36]	1345	32.7% (CNV 4.3%, SNV 94.3%, both 1.4%)	CES (trio and nontrio)	Cryptogenic (referral to genetic tests)	*AP4B1*, *SPAST*, *ATL1*, *REEP1*, *KIF1A*, *PLP1*, *RNASEH2B*, *TREX1*, *GNB1*, *GNAO1*, *PGK1*, *SPATA5*, *IFIH1*	Diagnostic yield: health-care-based control 10.5% Specific genomic locations of the identified CNVs were not detailed.
Takezawa et al. (2018) Japan [59]	17	52.9% (only SNV)	aCGH, trio-WES	Full-term CP without specific MRI findings	*CTNNB1*,*CYP2U1*,*SPAST*,*GNAO1*,*CACNA1A*,*AMPD2*, *STXBP1*, *SCN2A*	
Matthews et al. (2019) Canada [60]	50	65%	NGS	Atypical CP	*AKT3*, *ASXL1*, *ATP1A3*, *ATP8A2*, *CHRNA1*, *CSTB*, *DGKZ*, *EHMT1*, *EPHA4*, *GCDH*, *GNAO1*, *ITPA*, *KANK1*, *KCNJ6*, *KIDINS220*, *KMT2C*, *MECP2*, *NAA10*, *NBAS*, *PAK3*, *PALM*, *PLP1*, *PLXNA2*, *RANBP2*, *SCN3A*, *SPAST*, *TBCK*, *TCF4*, *TMEM67*, *TUBB4A*, *WDR45*	CMA-negative patients
Zouvelou et al. (2019) Greece [61]	47	48.9%	aCGH, MS-PCR, FMR1, TH, MLPA, CES	CP mimics	*ACTA1*, *ACTB*, *AMPD2*, *AP4M1*, *ATL1*, *ATP1A2*, *ATP1A3*, *ATP8A2*, *BSCL2*, *C12orf65*, *CASK*, *CERS1*, *CLCN2*, *COL4A1*, *COL4A2*, *CPA6*, *CPT1A*, *CPT2*, *CSF1R*, *CYP27A1*, *DAOA*, *DARS*, *DDC*, *DMD*, *DYNC1H1*, *EDA*, *EIF2B5*, *EIF4A2*, *ELOVL4*, *ERCC6*, *FA2H*, *FARS2*, *FBXO7*, *FGF14*, *FLNA*, *FOXP1*, *GAD1*, *GAD2*, *GATM*, *GBA*, *GCH1*, *GLRA1*, *GLRB*, *GNAO1*, *GNB1*, *GPR56*, *GRIN1*, *GRIN2A*, *GRIN2B*, *HADHA*, *HADHB*, *HARS*, *HNRNPU*, *HSPB1*, *HSPB8*, *HTRA2*, *IARS2*, *IGHMBP2*, *IKBKG*, *INF2*, *ISCU*, *KCNA1*, *KCNC3*, *KCNJ10*, *KCNQ2*, *KIF1A*, *KIF5A*, *KMT2B*, *L1CAM*, *LAMA2*, *LAMP2*, *LARGE*, *LARS2*, *LMNA*, *LRRK2*, *MARS2*, *MECP2*, *MFN2*, *and MOBP*	Risk factor: epilepsy
Nejabat et al. (2021) Iran [62]	66	45.2% (only SNV)	WES	Atypical	*HACE1*, *SPEG*, *SLC13A5*, *TRAPPC4*, *FBXL4*, *TDP2*, *GAMT*, *LAMB1*, *OCLN*, *WWOX*, *TREX1*, *SURF1*, *WDR45*, *LAMA2*, *MTHFR*, *MOCS1*, *DOM1*, *SEPSECS*, *GABRB1*, *KCNT1*, *AP4M1*, *PDHX*, *FOXG1*, *ATP6V1A*, *SPR*, *PIGG*, *ATL1*, *SNX14*	
Rosello et al. (2021) Spain [63]	20	55% (only SNV)	CMA, trio-CES	Idiopathic CP	*AP4B1*, *IFIH1*, *SPAST*, *ATL1*, *PGK1*, *SPATA5*, *GNAO1*, *GNB1*, *RNASEH2B*	Sporadic 64% Risk factors: spastic quadriplegia, epilepsy, gross motor impairment
Chopra et al. (2022) United States of America [64]	24	29% (only SNV)	WES (trio and nontrio)	Cryptogenic	*ECHS1*, *SATB2*, *ZMYM2*, *ADAT3*, *COL4A1*, *THOC2*, *SLC16A2*, *SPAST*, *POLR2A*, *GNAO1*, *PDHX*, *ACADM*, *ATL1*	Diagnostic yield: CP masquerader 60% (3/5)
Li et al. (2022) [65]	66	45% (only SNV)	WES	Idiopathic CP	*SPAST*, *KIF1A*, *COL4A1*, *BCL11A*, *BCL11B*, *TUBA1A*, *TUBB2B*, *ATL1*, *RARS2*, *PTK7*, *TARS*, *TYW1*, *GPAM*	
Yechieli et al. (2022) Israel [66]	45	58% (CNV 18%, SNV 40%)	CMA, trio-WES	Cryptogenic CP	15q11.2 microdeletion, 17p11.2 duplication, 12p13.33-p11.23 duplication, and 18p11.32 deletion *AIFM1*, *ELOVL1*, *VPS11*, *SYNE1*, *MAPK8IP3*, *PCDH19*, *AP2M1*, *PHF8*, *ARHGEF10*	Sporadic 62% Risk factors: associated comorbidities
Our study	10	90% (CNV 30%, SNV 60%)	Karyotyping, CMA, WES, WGS (trio and nontrio)	Atypical CP	15p11.2 microdeletion, 17p11.2 duplication, 12p13.33p11.23 duplication, and 18p11.32 microdeletion *SLC2A1*, *PLAA*, *CDC42BPB*, *CACNA1D*, *ALG12*, *SACS*	Sporadic 40%

SNV, single nucleotide variation; CNV, copy number variation; NGS, next-generation sequencing; aCGH, array comparative genomic hybridization; WES, whole exome sequencing; MS-PCR, methylation-specific PCR; MLPA, multiplex ligation-dependent probe amplification; CES, clinical exome sequencing; CMA, chromosomal microarray; WGS, whole genome sequencing.

## Data Availability

The data are contained within this article.

## References

[1] Rosenbaum P., Paneth N., Leviton A., Goldstein M., Bax M., Damiano D., Dan B., Jacobsson B. (2007). A report: The definition and classification of cerebral palsy April 2006. Dev. Med. Child Neurol. Suppl..

[2] Colver A., Fairhurst C., Pharoah P.O. (2014). Cerebral palsy. Lancet.

[3] MacLennan A.H., Thompson S.C., Gecz J. (2015). Cerebral palsy: Causes, pathways, and the role of genetic variants. Am. J. Obstet. Gynecol..

[4] McIntyre S., Morgan C., Walker K., Novak I. (2011). Cerebral palsy—Don’t delay. Dev. Disabil. Res. Rev..

[5] McIntyre S., Goldsmith S., Webb A., Ehlinger V., Hollung S.J., McConnell K., Arnaud C., Smithers-Sheedy H., Oskoui M., Khandaker G. (2022). Global prevalence of cerebral palsy: A systematic analysis. Dev. Med. Child Neurol..

[6] Maudsley G., Hutton J.L., Pharoah P.O. (1999). Cause of death in cerebral palsy: A descriptive study. Arch. Dis. Child..

[7] Moreno-De-Luca A., Ledbetter D.H., Martin C.L. (2012). Genetic insights into the causes and classification of the cerebral palsies. Lancet Neurol..

[8] McMichael G., Bainbridge M.N., Haan E., Corbett M., Gardner A., Thompson S., van Bon B.W., van Eyk C.L., Broadbent J., Reynolds C. (2015). Whole-exome sequencing points to considerable genetic heterogeneity of cerebral palsy. Mol. Psychiatry.

[9] Leach E.L., Shevell M., Bowden K., Stockler-Ipsiroglu S., van Karnebeek C.D. (2014). Treatable inborn errors of metabolism presenting as cerebral palsy mimics: Systematic literature review. Orphanet J. Rare Dis..

[10] Fahey M.C., Maclennan A.H., Kretzschmar D., Gecz J., Kruer M.C. (2017). The genetic basis of cerebral palsy. Dev. Med. Child Neurol..

[11] Boycott K.M., Rath A., Chong J.X., Hartley T., Alkuraya F.S., Baynam G., Brookes A.J., Brudno M., Carracedo A., den Dunnen J.T. (2017). International Cooperation to Enable the Diagnosis of All Rare Genetic Diseases. Am. J. Hum. Genet..

[12] Tarailo-Graovac M., Shyr C., Ross C.J., Horvath G.A., Salvarinova R., Ye X.C., Zhang L.H., Bhavsar A.P., Lee J.J., Drögemöller B.I. (2016). Exome Sequencing and the Management of Neurometabolic Disorders. N. Engl. J. Med..

[13] Novak I., Morgan C., Adde L., Blackman J., Boyd R.N., Brunstrom-Hernandez J., Cioni G., Damiano D., Darrah J., Eliasson A.C. (2017). Early, Accurate Diagnosis and Early Intervention in Cerebral Palsy: Advances in Diagnosis and Treatment. JAMA Pediatr..

[14] Korzeniewski S.J., Slaughter J., Lenski M., Haak P., Paneth N. (2018). The complex aetiology of cerebral palsy. Nat. Rev. Neurol..

[15] Hollung S.J., Bakken I.J., Vik T., Lydersen S., Wiik R., Aaberg K.M., Andersen G.L. (2020). Comorbidities in cerebral palsy: A patient registry study. Dev. Med. Child Neurol..

[16] McGuire D.O., Tian L.H., Yeargin-Allsopp M., Dowling N.F., Christensen D.L. (2019). Prevalence of cerebral palsy, intellectual disability, hearing loss, and blindness, National Health Interview Survey, 2009–2016. Disabil. Health J..

[17] Berger S.M., Bartsch D. (2014). The role of L-type voltage-gated calcium channels Cav1.2 and Cav1.3 in normal and pathological brain function. Cell Tissue Res..

[18] Krägeloh-Mann I., Horber V. (2007). The role of magnetic resonance imaging in elucidating the pathogenesis of cerebral palsy: A systematic review. Dev. Med. Child Neurol..

[19] Srivastava S., Love-Nichols J.A., Dies K.A., Ledbetter D.H., Martin C.L., Chung W.K., Firth H.V., Frazier T., Hansen R.L., Prock L. (2019). Meta-analysis and multidisciplinary consensus statement: Exome sequencing is a first-tier clinical diagnostic test for individuals with neurodevelopmental disorders. Genet. Med..

[20] Lewis S.A., Shetty S., Wilson B.A., Huang A.J., Jin S.C., Smithers-Sheedy H., Fahey M.C., Kruer M.C. (2020). Insights from Genetic Studies of Cerebral Palsy. Front. Neurol..

[21] Gonzalez-Mantilla P.J., Hu Y., Myers S.M., Finucane B.M., Ledbetter D.H., Martin C.L., Moreno-De-Luca A. (2023). Diagnostic Yield of Exome Sequencing in Cerebral Palsy and Implications for Genetic Testing Guidelines: A Systematic Review and Meta-analysis. JAMA Pediatr..

[22] Cox D.M., Butler M.G. (2015). The 15q11.2 BP1-BP2 microdeletion syndrome: A review. Int. J. Mol. Sci..

[23] Han J.Y., Park J. (2021). Phenotypic Diversity of 15q11.2 BP1–BP2 Deletion in Three Korean Families with Development Delay and/or Intellectual Disability: A Case Series and Literature Review. Diagnostics.

[24] Grama A., Sîrbe C., Miclea D., Cǎinap S.S., Huniadi D., Bulata B., Pop T.L. (2021). Case Report: Potocki-Lupski Syndrome in Five Siblings. Front. Pediatr..

[25] Ciaccio C., Pantaleoni C., Milani D., Alfei E., Sciacca F.L., Canafoglia L., Erbetta A., D’Arrigo S. (2020). Neurological phenotype of Potocki-Lupski syndrome. Am. J. Med. Genet. A.

[26] Shuib S., Saaid N.N., Zakaria Z., Ismail J., Abdul Latiff Z. (2017). Duplication 17p11.2 (Potocki-Lupski Syndrome) in a child with developmental delay. Malays. J. Pathol..

[27] Oliveira J.S., Joaquim T.M., Silva R., Souza D.H., Martelli L.R., Moretti-Ferreira D. (2020). Non-mosaic partial duplication 12p in a patient with dysmorphic characteristics and developmental delay. Genet. Mol. Biol..

[28] Halperin R., Arnon L., Nasirov S., Friedensohn L., Gershinsky M., Telerman A., Friedman E., Bernstein-Molho R., Tirosh A. (2023). Germline CDKN1B variant type and site are associated with phenotype in MEN4. Endocr.-Relat. Cancer.

[29] Poirsier C., Landais E., Bednarek N., Nobecourt J.M., Khoury M., Schmidt P., Morville P., Gruson N., Clomes S., Michel N. (2014). Report on 3 patients with 12p duplication including GRIN2B. Eur. J. Med. Genet..

[30] Wang F., Ning S., Yu B., Wang Y. (2021). USP14: Structure, Function, and Target Inhibition. Front. Pharmacol..

[31] Kantaputra P.N., Limwongse C., Tochareontanaphol C., Mutirangura A., Mevatee U., Praphanphoj V. (2006). Contiguous gene syndrome of holoprosencephaly and hypotrichosis simplex: Association with an 18p11.3 deletion. Am. J. Med. Genet. A.

[32] Verrotti A., Palka C., Prezioso G., Alfonsi M., Calabrese G., Palka G., Chiarelli F. (2015). Deletion 18p11.32p11.31 in a Child with Global Developmental Delay and Atypical, Drug-Resistant Absence Seizures. Cytogenet. Genome Res..

[33] Srivastava S., Cohen J.S., Vernon H., Barañano K., McClellan R., Jamal L., Naidu S., Fatemi A. (2014). Clinical whole exome sequencing in child neurology practice. Ann. Neurol..

[34] Finucane B.M., Myers S.M., Martin C.L., Ledbetter D.H. (2020). Long overdue: Including adults with brain disorders in precision health initiatives. Curr. Opin. Genet. Dev..

[35] Sanchez Fernandez I., Loddenkemper T., Gainza-Lein M., Sheidley B.R., Poduri A. (2019). Diagnostic yield of genetic tests in epilepsy: A meta-analysis and cost-effectiveness study. Neurology.

[36] Moreno-De-Luca A., Millan F., Pesacreta D.R., Elloumi H.Z., Oetjens M.T., Teigen C., Wain K.E., Scuffins J., Myers S.M., Torene R.I. (2021). Molecular Diagnostic Yield of Exome Sequencing in Patients With Cerebral Palsy. JAMA.

[37] Cooper M.S., Fahey M.C., Mackay M.T. (2022). Making waves: The changing tide of cerebral palsy. J. Paediatr. Child Health.

[38] Koch H., Weber Y.G. (2019). The glucose transporter type 1 (Glut1) syndromes. Epilepsy Behav..

[39] Leen W.G., Klepper J., Verbeek M.M., Leferink M., Hofste T., van Engelen B.G., Wevers R.A., Arthur T., Bahi-Buisson N., Ballhausen D. (2010). Glucose transporter-1 deficiency syndrome: The expanding clinical and genetic spectrum of a treatable disorder. Brain.

[40] Gras D., Roze E., Caillet S., Méneret A., Doummar D., Billette de Villemeur T., Vidailhet M., Mochel F. (2014). GLUT1 deficiency syndrome: An update. Rev. Neurol..

[41] Papadopoulos C., Kirchner P., Bug M., Grum D., Koerver L., Schulze N., Poehler R., Dressler A., Fengler S., Arhzaouy K. (2017). VCP/p97 cooperates with YOD1, UBXD1 and PLAA to drive clearance of ruptured lysosomes by autophagy. EMBO J..

[42] Zhang F., Sha J., Wood T.G., Galindo C.L., Garner H.R., Burkart M.F., Suarez G., Sierra J.C., Agar S.L., Peterson J.W. (2008). Alteration in the activation state of new inflammation-associated targets by phospholipase A2-activating protein (PLAA). Cell. Signal..

[43] Hall E.A., Nahorski M.S., Murray L.M., Shaheen R., Perkins E., Dissanayake K.N., Kristaryanto Y., Jones R.A., Vogt J., Rivagorda M. (2017). PLAA Mutations Cause a Lethal Infantile Epileptic Encephalopathy by Disrupting Ubiquitin-Mediated Endolysosomal Degradation of Synaptic Proteins. Am. J. Hum. Genet..

[44] Falik Zaccai T.C., Savitzki D., Zivony-Elboum Y., Vilboux T., Fitts E.C., Shoval Y., Kalfon L., Samra N., Keren Z., Gross B. (2017). Phospholipase A2-activating protein is associated with a novel form of leukoencephalopathy. Brain.

[45] Dai C., Zeng S., Tan Z., Yang X., Du J., Lu G., Wang J. (2019). Neurodevelopmental disorder with progressive microcephaly, spasticity, and brain anomalies in China caused by novel mutations of PLAA. Clin. Genet..

[46] Heikkila T., Wheatley E., Crighton D., Schroder E., Boakes A., Kaye S.J., Mezna M., Pang L., Rushbrooke M., Turnbull A. (2011). Co-crystal structures of inhibitors with MRCKβ, a key regulator of tumor cell invasion. PLoS ONE.

[47] Chilton I., Okur V., Vitiello G., Selicorni A., Mariani M., Goldenberg A., Husson T., Campion D., Lichtenbelt K.D., van Gassen K. (2020). De novo heterozygous missense and loss-of-function variants in *CDC42BPB* are associated with a neurodevelopmental phenotype. Am. J. Med. Genet. A.

[48] Bock G., Gebhart M., Scharinger A., Jangsangthong W., Busquet P., Poggiani C., Sartori S., Mangoni M.E., Sinnegger-Brauns M.J., Herzig S. (2011). Functional properties of a newly identified C-terminal splice variant of Cav1.3 L-type Ca^2+^ channels. J. Biol. Chem..

[49] Scholl U.I., Goh G., Stölting G., de Oliveira R.C., Choi M., Overton J.D., Fonseca A.L., Korah R., Starker L.F., Kunstman J.W. (2013). Somatic and germline *CACNA1D* calcium channel mutations in aldosterone-producing adenomas and primary aldosteronism. Nat. Genet..

[50] Ortner N.J., Kaserer T., Copeland J.N., Striessnig J. (2020). De novo *CACNA1D* Ca^2+^ channelopathies: Clinical phenotypes and molecular mechanism. Pflugers Arch..

[51] Marquardt T., Denecke J. (2003). Congenital disorders of glycosylation: Review of their molecular bases, clinical presentations and specific therapies. Eur. J. Pediatr..

[52] Chantret I., Dupré T., Delenda C., Bucher S., Dancourt J., Barnier A., Charollais A., Heron D., Bader-Meunier B., Danos O. (2002). Congenital disorders of glycosylation type Ig is defined by a deficiency in dolichyl-P-mannose: Man7GlcNAc2-PP-dolichyl mannosyltransferase. J. Biol. Chem..

[53] Eklund E.A., Newell J.W., Sun L., Seo N.S., Alper G., Willert J., Freeze H.H. (2005). Molecular and clinical description of the first US patients with congenital disorder of glycosylation Ig. Mol. Genet. Metab..

[54] Kranz C., Basinger A.A., Güçsavaş-Çalıkoğlu M., Sun L., Powell C.M., Henderson F.W., Aylsworth A.S., Freeze H.H. (2007). Expanding spectrum of congenital disorder of glycosylation Ig (CDG-Ig): Sibs with a unique skeletal dysplasia, hypogammaglobulinemia, cardiomyopathy, genital malformations, and early lethality. Am. J. Med. Genet. Part A.

[55] Bouhlal Y., Amouri R., El Euch-Fayeche G., Hentati F. (2011). Autosomal recessive spastic ataxia of Charlevoix–Saguenay: An overview. Park. Relat. Disord..

[56] Aly K.A., Moutaoufik M.T., Zilocchi M., Phanse S., Babu M. (2022). Insights into *SACS* pathological attributes in autosomal recessive spastic ataxia of Charlevoix-Saguenay (AR*SACS*). Curr. Opin. Chem. Biol..

[57] Lessard I., Côté I., St-Gelais R., Hébert L.J., Brais B., Mathieu J., Rodrigue X., Gagnon C. (2024). Natural history of autosomal recessive spastic ataxia of Charlevoix-Saguenay: A 4-year longitudinal study. Cerebellum.

[58] Scaravilli A., Negroni D., Senatore C., Ugga L., Cosottini M., Ricca I., Bender B., Traschütz A., Başak A.N., Vural A. (2024). MRI-ARSACS: An Imaging Index for Autosomal Recessive Spastic Ataxia of Charlevoix-Saguenay (AR*SACS*) Identification Based on the Multicenter PROSPAX Study. Mov. Disord..

[59] Takezawa Y., Kikuchi A., Haginoya K., Niihori T., Numata-Uematsu Y., Inui T., Yamamura-Suzuki S., Miyabayashi T., Anzai M., Suzuki-Muromoto S. (2018). Genomic analysis identifies masqueraders of full-term cerebral palsy. Ann. Clin. Transl. Neurol..

[60] Matthews A.M., Blydt-Hansen I., Al-Jabri B., Andersen J., Tarailo-Graovac M., Price M., Selby K., Demos M., Connolly M., Drögemoller B. (2019). Atypical cerebral palsy: Genomics analysis enables precision medicine. Genet. Med..

[61] Zouvelou V., Yubero D., Apostolakopoulou L., Kokkinou E., Bilanakis M., Dalivigka Z., Nikas I., Kollia E., Perez-Dueñas B., Macaya A. (2019). The genetic etiology in cerebral palsy mimics: The results from a Greek tertiary care center. Eur. J. Paediatr. Neurol..

[62] Nejabat M., Inaloo S., Sheshdeh A.T., Bahramjahan S., Sarvestani F.M., Katibeh P., Nemati H., Tabei S.M.B., Faghihi M.A. (2021). Genetic Testing in Various Neurodevelopmental Disorders Which Manifest as Cerebral Palsy: A Case Study From Iran. Front. Pediatr..

[63] Rosello M., Caro-Llopis A., Orellana C., Oltra S., Alemany-Albert M., Marco-Hernandez A.V., Monfort S., Pedrola L., Martinez F., Tomás M. (2021). Hidden etiology of cerebral palsy: Genetic and clinical heterogeneity and efficient diagnosis by next-generation sequencing. Pediatr. Res..

[64] Chopra M., Gable D.L., Love-Nichols J., Tsao A., Rockowitz S., Sliz P., Barkoudah E., Bastianelli L., Coulter D., Davidson E. (2022). Mendelian etiologies identified with whole exome sequencing in cerebral palsy. Ann. Clin. Transl. Neurol..

[65] Li N., Zhou P., Tang H., He L., Fang X., Zhao J., Wang X., Qi Y., Sun C., Lin Y. (2022). In-depth analysis reveals complex molecular aetiology in a cohort of idiopathic cerebral palsy. Brain.

[66] Yechieli M., Gulsuner S., Ben-Pazi H., Fattal A., Aran A., Kuzminsky A., Sagi L., Guttman D., Schneebaum Sender N., Gross-Tsur V. (2022). Diagnostic yield of chromosomal microarray and trio whole exome sequencing in cryptogenic cerebral palsy. J. Med. Genet..

[67] Gupta R., Appleton R.E. (2001). Cerebral palsy: Not always what it seems. Arch. Dis. Child..

[68] Pham R., Mol B.W., Gecz J., MacLennan A.H., MacLennan S.C., Corbett M.A., van Eyk C.L., Webber D.L., Palmer L.J., Berry J.G. (2020). Definition and diagnosis of cerebral palsy in genetic studies: A systematic review. Dev. Med. Child Neurol..

[69] Kearney H.M., Thorland E.C., Brown K.K., Quintero-Rivera F., South S.T. (2011). American College of Medical Genetics standards and guidelines for interpretation and reporting of postnatal constitutional copy number variants. Genet. Med..

[70] Richards S., Aziz N., Bale S., Bick D., Das S., Gastier-Foster J., Grody W.W., Hegde M., Lyon E., Spector E. (2015). Standards and guidelines for the interpretation of sequence variants: A joint consensus recommendation of the American College of Medical Genetics and Genomics and the Association for Molecular Pathology. Genet. Med..

[71] Pejaver V., Byrne A.B., Feng B.J., Pagel K.A., Mooney S.D., Karchin R., O’Donnell-Luria A., Harrison S.M., Tavtigian S.V., Greenblatt M.S. (2022). Calibration of computational tools for missense variant pathogenicity classification and ClinGen recommendations for PP3/BP4 criteria. Am. J. Hum. Genet..

